# Supplementary material for: Development of a computer-aided design software for the quantitative evaluation of aesthetic damage
Source: PLoS One. 2019 Dec 18;14(12):e0226322. doi: 10.1371/journal.pone.0226322 (PMC6919621; doi:10.1371/journal.pone.0226322)
Supplement: S1 Code site — 1. (ZIP) [file pone.0226322.s001.zip › [EESC_jr][Projeto]Relatorio_07_21/reports/templates_zip/formwizard-master.zip!/formwizard-master/examples/example_8_multiple_wizards.html]

xml version="1.0" encoding="UTF-8"?


JQuery Form Wizard


### Example of a multiple wizards on the same page with browser back and forward buttons enabled

- Multiple wizards.
- BBQ plugin enabled via the historyEnabled option for both wizards.

---


First step - Name  
First name  
  
Surname

Step 2 - Personal information  
Social Security Number  


 -
  
Phone number  
 -
 -
  
\*Email

Last step - Username  
User name  
  
Password  
  
Retype password


---

First step - Name  
First name  
  
Surname  
  

Step 2 - Personal information  
Social Security Number  


 -
  
Phone number  
 -
 -
  
\*Email  
  

Last step - Username  
User name  
  
Password  
  
Retype password


---
